# Peculiar Cold-Induced Leukoagglutination in Mycoplasma pneumoniae Pneumonia

**DOI:** 10.4274/tjh.2017.0203

**Published:** 2017-12-01

**Authors:** Yasushi Kubota, Yuka Hirakawa, Kazuo Wakayama, Shinya Kimura

**Affiliations:** 1 Saga University Faculty of Medicine, Department of Internal Medicine, Division of Hematology, Saga, Japan; 2 Saga University Faculty of Medicine, Department of Transfusion Medicine, Saga, Japan; 3 Saga University Faculty of Medicine, Department of General Medicine, Saga, Japan; 4 Saga University Faculty of Medicine, Department of Clinical Laboratory Medicine, Saga, Japan

**Keywords:** Leukoagglutination, Cold agglutinin, Mycoplasma pneumoniae, Eosinophilia, Pseudoleukopenia

An 18-year-old woman was diagnosed with atypical pneumonia and treated with oral levofloxacin. Skin eruptions also appeared. On day 6 after admission, laboratory tests revealed the following: red blood cells (RBCs), 1.76x109/L; hemoglobin, 128 g/L; white blood cells (WBCs), 7x109/L with 56% neutrophils, 27% lymphocytes, 6% monocytes, 10.5% eosinophils, and 1% basophils. A peripheral blood smear showed not only RBC agglutination but also neutrophil aggregates, eosinophil aggregates, and monocyte aggregates (Figure 1). After warming to 37 °C, the agglutination disappeared. The RBC and WBC counts returned to 4.44x109/L and 9x109/L with 55% neutrophils, 26% lymphocytes, 6% monocytes, 12% eosinophils, and 1% basophils. Blood chemistry analysis showed total bilirubin of 0.4 mg/dL and lactate dehydrogenase of 510 U/L. A direct antiglobulin test showed 1+ anti-C3d and 1+ anti-C3b3d. A passive agglutination test in paired serum samples revealed seroconversion of M. pneumoniae antibodies (1:80 to 1:20,480). Cold agglutinin was detected to a titer of 1:8192.

Cold-induced erythrocyte agglutination is frequently observed in cases of M. pneumoniae infection, but leukoagglutination is rare [1,2]. Though the pathomechanism of leukoagglutination is still uncertain [3], it has been postulated that immunoglobulin M cold agglutinin directed against I antigens of the leukocyte membranes is responsible for transient cold-induced leukoagglutination [4]. A previous series of four pediatric cases of M. pneumoniae infection, all of which showed leukoagglutination, reported that eruption, eosinophilia, a high titer of cold agglutinin, and a high titer of M. pneumoniae antibodies were observed [5]. When leukocytopenia occurs in patients with these symptoms, pseudoleukopenia induced by leukoagglutination should be recognized as one potential cause.

## Figures and Tables

**Figure 1:**
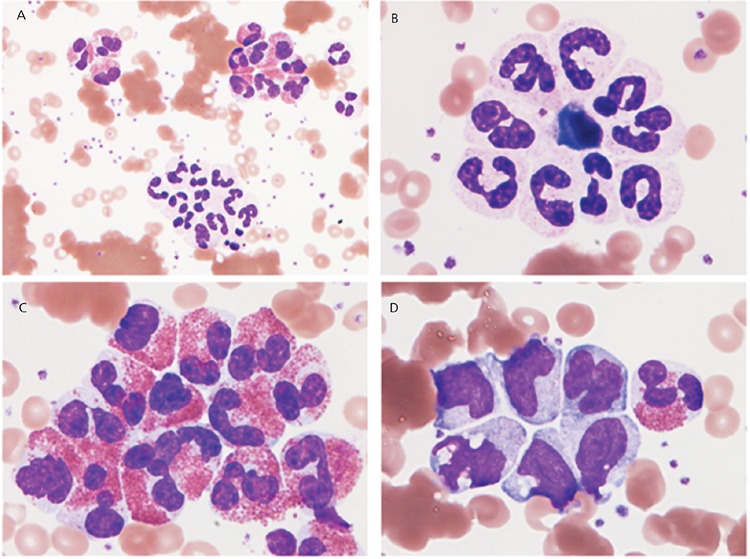
A peripheral blood smear showed not only RBC agglutination (A) but also neutrophil aggregates, eosinophil aggregates, and monocyte aggregates (A-D).
